# Estimating the Burden of Tuberculosis among Foreign-Born Persons Acquired Prior to Entering the U.S., 2005–2009

**DOI:** 10.1371/journal.pone.0027405

**Published:** 2011-11-29

**Authors:** Philip M. Ricks, Kevin P. Cain, John E. Oeltmann, J. Steve Kammerer, Patrick K. Moonan

**Affiliations:** Division of Tuberculosis Elimination, United States Centers for Disease Control and Prevention, Atlanta, Georgia, United States of America; St. Petersburg Pasteur Institute, Russian Federation

## Abstract

**Background:**

The true burden of reactivation of remote latent tuberculosis infection (reactivation TB) among foreign-born persons with tuberculosis (TB) within the United States is not known. Our study objectives were to estimate the proportion of foreign-born persons with TB due reactivation TB and to describe characteristics of foreign-born persons with reactivation TB.

**Methods:**

We conducted a cross-sectional study of patients with an *M. tuberculosis* isolate genotyped by the U.S. National TB Genotyping Service, 2005–2009. TB cases were attributed to reactivation TB if they were not a member of a localized cluster of cases. Localized clusters were determined by a spatial scan statistic of cases with isolates with matching TB genotype results. Crude odds ratios and 95% confidence intervals were used to assess relations between reactivation TB and select factors among foreign-born persons.

**Main Results:**

Among the 36,860 cases with genotyping and surveillance data reported, 22,151 (60%) were foreign-born. Among foreign-born persons with TB, 18,540 (83.7%) were attributed to reactivation TB. Reactivation TB among foreign-born persons was associated with increasing age at arrival, incidence of TB in the country of origin, and decreased time in the U.S. at the time of TB diagnosis.

**Conclusions:**

Four out of five TB cases among foreign-born persons can be attributed to reactivation TB and present the largest challenge to TB elimination in the U.S. TB control strategies among foreign-born persons should focus on finding and treating latent tuberculosis infection prior to or shortly after arrival to the United States and on reducing the burden of LTBI through improvements in global TB control.

## Introduction

In the United States, the proportion of reported tuberculosis (TB) cases among foreign-born persons doubled from 29% in 1993 to 59% in 2009, concurrent with a 54% decrease in total TB cases from 25,107 to 11,545 [1]. The TB incidence rate among foreign-born persons, at 18.7 per 100,000 in 2009, is nearly ten times the TB incidence among the U.S.-born population. These disparities between U.S. and foreign-born persons have raised concerns about the ability to further reduce, and eventually eliminate TB in the United States [2], [3]. Similar trends were observed in other low-incidence countries globally [4]–[6], prompting the World Health Organization to suggest that when the proportion of foreign-born cases exceeds 70% of the national total of reported cases, no greater than 2% decrease in annual TB incidence can be expected without rethinking contemporary TB prevention and control strategies [7].

There are three main TB control strategies: 1) finding and treating patients with TB disease as quickly as possible, 2) examining persons in close contact with a person diagnosed with infectious TB disease, in order to diagnose and treat TB disease and latent TB infection (LTBI), and 3) finding and treating people with LTBI to prevent TB disease [8]. Although most TB among the foreign-born has been attributed to reactivation of remotely acquired tuberculosis infection, or reactivation TB (most likely acquired in their country of origin), control measures to detect and treat LTBI are not widely implemented [9], [10]. Understanding the epidemiology of reactivation TB will assist in planning TB control efforts among foreign-born persons [11]–[13], without which the goal of TB elimination will not be possible [3], [12].

TB genotyping results, when combined with epidemiologic data, may help identify recent TB transmission [14]. A basic assumption of this approach is that recent TB transmission is localized in place and time, that is, resulting from progression to TB disease from an infection acquired a short time ago and in the same location. Reactivation TB is a result of remotely acquired TB infection that is generally associated with progression of disease from an infection acquired in the past and often in another location. Thus cases with TB genotypes that are not clustered in time and space are often used as a marker of remotely acquired TB infection [14]. Researchers using TB genotyping have reported that most cases among foreign-born persons are due to the reactivation of remotely acquired TB infection occurring before U.S. arrival [15]–[19]. However, these studies were small or geographically limited.

In 2004, the U.S. Centers for Disease Control and Prevention (CDC) initiated national TB genotyping surveillance to routinely characterize at least one *M. tuberculosis* complex isolate from every TB case in the United States [20]. Data collected from this system offers a unique opportunity to describe the molecular epidemiology of tuberculosis on a national level. The purpose of this analysis is to estimate the proportion of tuberculosis in the United States attributable to reactivation of remotely acquired TB infection and to assess key factors associated with reactivation TB comparing U.S. and foreign-born persons.

## Methods

### Study Design and Participants

We conducted a cross-sectional analysis of culture-confirmed TB reported to the U.S. National Tuberculosis Surveillance System (NTSS) by the 50 states and the District of Columbia from January 2005 to December 2009. Clinical, demographic, and epidemiologic variables for each case are collected for surveillance purposes and are described elsewhere [1]. Consistent with U.S. Census Bureau definitions, individuals were classified as foreign-born if they were not born in the United States or an associated jurisdiction, or if they were born in a foreign country and neither parent was a U.S. citizen [21]. All others were classified as U.S.-born.

*M. tuberculosis* complex isolates were characterized using a standardized protocol for spacer oligonucleotide typing (spoligotyping) and using a panel of 12 mycobacterial interspersed repetitive unit variable number of tandem repeats (MIRU–VNTR) loci [22]–[24]. Genotyping results for each submitted isolate were linked to NTSS case records by state and local TB control programs using a standardized case identification number [1] and a unique laboratory accession number to form discrete individual isolate-case records [25]. Our final study population included all reported culture-positive TB cases reported from January 2005 to December 2009 with a complete spoligotype and 12-locus MIRU–VNTR result.

Genotype clusters were defined as at least 2 cases with matching spoligotype and 12-locus MIRU-VNTR results reported within statistically significant geospatial zones determined by a spatial scan statistic as described elsewhere [26]. Briefly, three scans comprised of 3-year overlapping intervals (Scan A: 2005–2007; Scan B: 2006–2008; Scan C: 2007–2009) were performed to identify spatial clusters occurring within a 3-year period. If cases were identified as a member of a statistically significant spatial cluster in any of the 3 periods, they were considered clustered. There was no duplicative case counting. All cases that were not genotypically and spatially clustered were considered reactivation of remotely acquired TB infection, or reactivation TB.

### Data Analysis

We used a Pearson's chi-square statistic to assess differences in proportions of reactivation TB between U.S. and foreign-born persons. We calculated the proportions of foreign born cases attributed to reactivation TB for each of the 25 countries that contribute the largest proportions of reactivation TB. Crude odds ratios and 95% confidence intervals were used to evaluate the relationship between select factors (annual TB incidence in country of origin, age at arrival in the U.S., and time in the U.S. after arrival) and reactivation TB among foreign-born persons.

We grouped the following continuous variables into three or more ordinal categories: age at arrival in U.S., time since arrival in U.S. to TB diagnosis, and TB incidence rate in country of origin. Time since arrival was categorized as <2 years, ≥2 years to <5 years, and ≥5 years. These categories were chosen to reflect the known risk of reactivation TB, which is highest in the first 2 years after infection (or after high-intensity exposure has ended) with rapid decline after 5 years [27]–[30]. TB incidence in a person's country of origin was categorized into three groups based on 2010 WHO estimates [31]: 150 or more cases per 100,000 population, 20 to 149 cases per 100,000, and less than 20 cases per 100,000.

## Results

**Figure 1:**
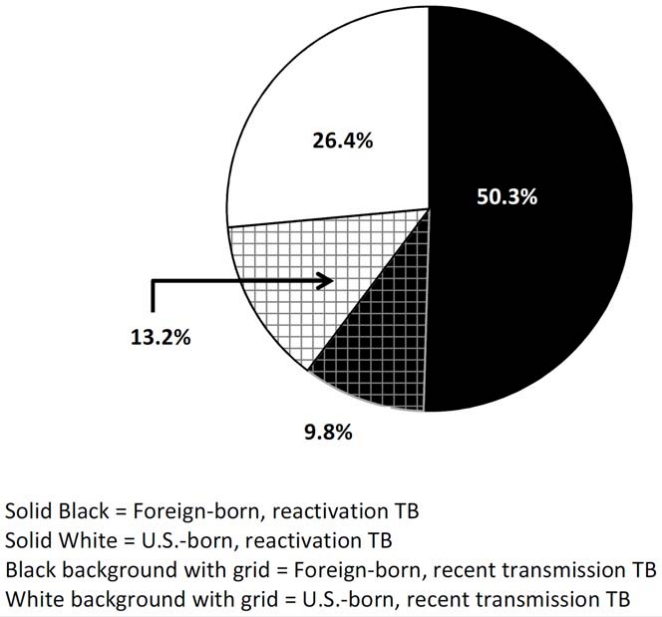
Tuberculosis attributed to recent transmission versus reactivation of remote tuberculosis infection by nativity, United States 2005–2009. Legend: Solid black = Foreign-born, reactivation TB Solid white = U.S.-born, reactivation TB Black background with grid = Foreign-born, recent transmission TB White background with grid = U.S.-born, recent transmission TB.

From 2005 to 2009, there were 65,529 new TB cases reported to the NTSS, of which 51,015 (78%) were culture-confirmed. Genotype and country of origin were available for 72% of all new culture-confirmed TB cases, providing a study sample of 36,860 cases; 22,151 (60.3%) among foreign-born persons and 14,594 (39.7%) among U.S.-born persons (Figure 1). A significantly higher proportion of TB cases were attributed to reactivation TB among foreign-born persons compared to U.S. born persons (83.4% vs. 66.6%, *p*<0.001). Among all cases, 18,540 (50.3%) were foreign-born attributable to reactivation TB; 3,611 (9.8%) were foreign-born attributable to recent transmission; 9,723 (26.4%) were U.S.-born attributable to reactivation; 4,871 (13.2%) were U.S.-born and attributable to recent transmission (Figure 1).

Mexico, the Philippines, and Viet Nam, were the top three countries of origin, accounting for 23.7%, 11.7%, and 8.7% of total foreign-born cases in the U.S., respectively (Table 1). Foreign-born persons from Bangladesh had the highest proportion of TB cases attributed to reactivation TB (97.6%) followed by India (95.2%) and Myanmar (93.3%) (Table 1).

**Table 1:** Top twenty-five countries of origin with the highest proportion of reactivation TB among foreign-born persons diagnosed with TB in United States, 2005–2009*.

	Reactivation TB		Recent TB Transmission		Total	
	n	(%)	n	(%)	n	(%)^1^
***Country***						
*Bangladesh*	120	(97.6)	3	(2.4)	123	(0.6)
*India*	1,503	(95.2)	76	(4.8)	1,579	(7.1)
*Myanmar*	166	(93.3)	12	(6.7)	178	(0.8)
*Pakistan*	215	(91.1)	21	(8.9)	236	(1.1)
*Kenya*	226	(90.4)	24	(9.6)	250	(1.1)
*Indonesia*	122	(90.4)	13	(9.6)	135	(0.6)
*Nepal*	159	(90.3)	17	(9.7)	176	(0.8)
*El Salvador*	360	(88.9)	45	(11.1)	405	(1.8)
*Cambodia*	252	(88.4)	33	(11.6)	285	(1.3)
*Republic of South Korea*	455	(88.2)	61	(11.8)	516	(2.3)
*Honduras*	397	(87.8)	55	(12.2)	452	(2.0)
*Thailand*	131	(86.8)	20	(13.2)	151	(0.7)
*Viet Nam*	1,661	(86.7)	255	(13.3)	1,916	(8.7)
*Haiti*	340	(85.2)	59	(14.8)	399	(1.8)
*People's Republic of China*	990	(84.7)	179	(15.3)	1,169	(5.3)
*Peru*	313	(83.0)	64	(17.0)	377	(1.7)
*Guatemala*	570	(82.8)	118	(17.2)	688	(3.1)
*Mexico*	4,289	(82.0)	943	(18.0)	5,232	(23.7)
*Ethiopia*	457	(81.3)	105	(18.7)	562	(2.5)
*Laos*	203	(81.2)	47	(18.8)	250	(1.1)
*Somalia*	393	(78.6)	107	(21.4)	500	(2.3)
*Nigeria*	95	(77.2)	17	(13.8)	123	(0.9)
*The Philippines*	1,959	(75.9)	623	(24.1)	2,582	(11.7)
*Ecuador*	243	(70.6)	101	(29.4)	344	(1.6)
*Dominican Republic*	124	(60.5)	81	(39.5)	205	(0.9)
**All Foreign-born**	**18,540**	**(83.7)**	**3,611**	**(16.3)**	**22,151**	

*Among countries with at least 100 reported cases.

1 Column percent.

Compared to foreign-born persons with TB from countries with TB incidence of <20 cases per 100,000, the odds of having reactivation TB were greatest among persons from countries with a TB incidence > = 150/100,000 (OR = 1.3; 95% CI = 1.2, 1.4).) (Table 2). Age at arrival in the United States was associated with reactivation TB. Compared to foreign-born persons less than 14 years of age, the odds of reactivation TB was 2.0 (95% CI: 1.9, 2.2) times more likely among those aged 15–24 years at arrival; 2.5 (95% CI: 2.3, 2.7) times more likely among those aged 25–44 years; 2.8 (95% CI: 2.5, 3.1) times more likely among those aged 45–64 years, and 3.2 (95%CI: 2.7, 3.8) times more likely among those >65 years (Table 3). Less time in the U.S. prior to TB diagnosis was also associated with increased odds of reactivation TB among foreign-born persons (Table 4). Compared to foreign-born persons in the U.S. more than 5 years, the odds of reactivation TB were 1.3 (95% CI: 1.2, 1.4) times higher among those in the U.S. for less than 2 years, and 1.1 (95% CI 1.0, 1.3) times higher among those in the U.S for 2 to 5 years before TB diagnosis (Table 4).

**Table 2:** Proportion of reactivation TB among foreign-born TB cases by estimated annual TB incidence in the country of origin, United States 2005–2009.

	Reactivation TB		Recent TB Transmission		Total		Odds Ratio	(95% CI)
	*n*	*(%)*	*n*	*(%)*	*n*	*(%)* 1		
***Estimated TB Incidence*** 2								
*<20*	4,788	(82.0)	1,060	(18.0)	5,848	(26.4)	*referent*	- -
*20 to 149*	4,792	(83.2)	967	(16.8)	5,759	(26.0)	1.1	(1.0, 1.2)
*>150*	8,960	(85.0)	1,584	(15.0)	10,544	(32.5)	1.3	(1.2, 1.4)

1 Column percent.

2 per 100,000 population.

**Table 3:** Proportion of reactivation TB among foreign-born TB cases by age at tuberculosis diagnosis, United States 2005–2009.

	Reactivation TB		Recent TB Transmission		Total		Odds Ratio	(95% CI)
	*n*	*(%)*	*n*	*(%)*	*n*	*(%)* 1		
***Age at tuberculosis diagnosis (in years)***								
*0*–*14*	141	(78.3)	39	(21.7)	180	(0.8)	*referent*	- -
*15*–*24*	2,697	(82.0)	594	(18.0)	3,291	(14.9)	2.1	(1.8, 2.5)
*25*–*44*	7,546	(84.1)	1,425	(15.9)	8,968	(40.5)	2.3	(2.0, 2.7)
*45*–*64*	4,620	(82.3)	992	(17.7)	5,612	(25.3)	1.9	(1.7, 2.3)
*≥65*	3,536	(86.3)	561	(15.5)	4,100	(18.5)	4.5	(3.7, 5.2)

1 Column percent.

**Table 4:** Proportion of reactivation TB among foreign-born TB cases by time in the U.S. prior to TB diagnosis, United States 2005–2009.

	Reactivation TB		Recent TB Transmission		Total		Odds Ratio^2^	(95% CI)
	*n*	*(%)*	*n*	*(%)*	*n*	*(%)* 1		
***Time in the United States prior to TB Diagnosis***								
*Less than 2 years*	6,575	(85.7)	1,110	(14.5)	7,675	(34.6)	1.3	(1.2, 1.4)
*2 to 5 years*	2,795	(84.0)	531	(16.0)	3,326	(15.0)	1.1	(1.0, 1.3)
*Greater than 5 years*	9,170	(82.2)	1,980	(17.8)	11,150	(50.3)	*referent*	- -

1 Column percent.

2 Comparing nonclustered to clustered.

## Discussion

Foreign-born persons account for the majority of cases reported in the U.S. [1] and approximately 4 out of 5 of these cases can be attributed to reactivation TB. Cases of reactivation TB among the foreign-born account for half of all cases and represent the single largest challenge to achieving TB elimination in the U.S. This national estimate is consistent with numerous smaller, more localized studies in the United States [16]–[19], [32] and in Europe [33], [34]. This evidence suggests that the majority of reported TB cases are due LTBI acquired prior to entering the U.S. and subsequent reactivation of TB among foreign-born persons, either occurring soon before or soon after entering the U.S. Therefore, addressing the burden of LTBI in persons entering the United States through either LTBI testing and treatment or through prevention of LTBI will be essential for TB elimination. While immigration screening programs have been enhanced to improve detection and treatment of tuberculosis disease in this population, the primary purpose is to identify active TB disease, not LTBI testing and treatment among adults [35].

Currently, there is no policy to test for LTBI among foreign-born adults prior to or during their entry process to the U.S. [36]. The only persons that are tested for LTBI during the immigration process are children aged 2–14 years from countries with TB incidence exceeding 20/100,000. The primary purpose of this testing is to identify children who need more complete evaluation for active TB disease, not to identify and treat patients with LTBI. For foreign-born persons who already reside in the United States, current guidelines recommend LTBI testing only for persons who have been in the U.S. <5 years [37].

To meet the goal of TB elimination in the U.S., (1 case per 1,000,000 persons per year), the burden of LTBI among the foreign-born will have to be reduced [3], [13]. There are two general approaches to doing this: 1) find and treat foreign-born persons with LTBI (either before or after they enter the United States); or 2) prevent LTBI in foreign-born persons by improving TB control in their country of origin, thereby reducing TB transmission and the risk of TB infection [38].

In addition to testing for LTBI, success also requires initiating and completing treatment of persons with LTBI. The current standard therapy for LTBI, isonaizid (INH), can reduce the risk of progression from LTBI to active TB by as much as 90% if taken daily for 9 months [39]. Unfortunately completion rates for 9 months of INH are poor, but vary under programmatic conditions; with many programs reporting less than 50% completion [39]. A new regimen, a once weekly dose of INH and rifapentine taken for 12 weeks (3HP), has the potential to improve adherence and completion of therapy [40], [41] and perhaps may influence the intent to treat LTBI among foreign-born persons. These regimens are likely to be efficacious, as only 9.4% (n = 1,748) of all foreign-born person with reactivation TB had isolates that were resistant to INH, rifampicin, or both during the 5 year study period.

This study does have some important limitations. First, isolate submission to the U.S. National TB Genotyping Service (NTGS) is voluntary, thus the database, although large, did not contain all reported culture-positive TB cases for the study period. However the potential for systematic bias due to voluntary isolate submission is unlikely, as we found no statistical difference with regards to clinical, demographic, or social factor between TB cases with and those without genotyping results (data not shown). Second, spatial and genotype clustering serves only as a proxy for differentiating recent TB transmission and reactivation TB in the absence of detailed data on interpersonal connections between cases. It is possible that recent immigrants who became infected with the same genotype elsewhere and resettle in the same neighborhood in the U.S. could subsequently develop TB after resettlement and falsely be considered recent TB transmission. Third, although spoligotyping and 12-locus MIRU-VNTR have good discriminatory power, these methods may not provide the resolution necessary to differentiate evolutionarily close strains [42]–[43]. Moreover, deriving the portion of reactivation TB among foreign-born persons based on genotyping methods is dependent on the genetic diversity of strains circulating in different regions of the world. The introduction of a more specific expanded panel of 24 MIRU-VNTR loci in 2009 to NTGS may reduce this misclassification in the future. It is important to note that each of these limitations would most likely underestimate the true proportion of foreign-born persons attributed to reactivation TB, further emphasizing the importance of reactivation TB in the United States.

At present, the high burden of reactivation TB is caused primarily by 1) the lack of LTBI screening among adult immigrants, both prior to arrival and after taking up residence, and 2) low initiation and completion rates for LTBI treatment [37]; and 3) ongoing high rates of TB disease (and therefore infection) in many countries from which foreign-born persons come to the United States. To reduce the incidence of TB among foreign-born persons, efforts to increase testing and for those testing positive both initiation and completion of therapy must be applied both for those planning to immigrate and those already residing in the U.S., and thus needs to be combined with efforts to improve global TB control. While universal testing and treatment for LTBI among all foreign-born persons would not be cost-effective, as most would not progress to active TB disease [30], targeted, cost-effective testing strategies need to be developed that account for incidence in country of origin, travel, and medical history [44]–[45]. Given the low specificity of TST, the use of interferon-gamma release assays (IGRA) might be a more suitable screening tool in these situations [46], [47]. Studies to measure the efficacy and cost-benefit of these programs should include foreign-born populations residing in the U.S. and those planning to immigrate, as failure to detect and treat LTBI in the country of origin can have costs in the U.S [46]. Strategies for completion of LTBI treatment among foreign-born persons and potential immigrants also need to be developed, which might include non-traditional DOT strategies, incentives, shortened treatment regimens, and social service support. Operations research would be needed to identify the strategy that is most feasible, effective, and acceptable.

Finally, efforts to improve global TB control are needed. TB incidence remains very high in many countries, causing a very high proportion of persons living in those countries to develop LTBI. If TB incidence could be brought down, LTBI could be prevented in many persons. All of these efforts combined will be needed to substantially impact the burden of LTBI in foreign-born persons entering the United States. Without addressing that LTBI burden, it will be impossible to achieve our goal of TB elimination.
